# Occurrence of Sinonasal Intestinal-Type Adenocarcinoma and Non-Intestinal-Type Adenocarcinoma in Two Countries with Different Patterns of Wood Dust Exposure

**DOI:** 10.3390/cancers13205245

**Published:** 2021-10-19

**Authors:** Ilmo Leivo, Reetta Holmila, Danièle Luce, Torben Steiniche, Michael Dictor, Pirjo Heikkilä, Kirsti Husgafvel-Pursiainen, Henrik Wolff

**Affiliations:** 1 Institute of Biomedicine, Pathology, University of Turku, Kiinamyllynkatu 10 D 5035, 20520 Turku, Finland; 2 Turku University Central Hospital, 20521 Turku, Finland; 3 Institute of Occupational Health, PB 40, 00251 Helsinki, Finland; reetta.holmila@gmail.com (R.H.); pirjo.heikkila@elisanet.fi (P.H.); kirsti.husgafvel@gmail.com (K.H.-P.); henrik.wolff@ttl.fi (H.W.); 4 Inserm U 1085-Institut de Recherche en Santé, Environnement et Travail (IRSET), Faculté de Médecine, BP-145, Campus de Fouillole, 97154 Pointe-à-Pitre, Guadeloupe, France; daniele.luce@inserm.fr; 5 Institute of Pathology, Aarhus University Hospital, Palle Juul-Jensens Boulevard 99, 8200 Aarhus, Denmark; steiniche@clin.au.dk; 6 Department of Pathology, Lund Hospital, Sölvegatan 25B, 221 85 Lund, Sweden; michael.dictor@lu.se; 7 Department of Pathology, University of Helsinki, PB 20, 00014 Helsinki, Finland

**Keywords:** immunohistochemistry, sinonasal adenocarcinoma, sinonasal intestinal-type adenocarcinoma, sinonasal non-intestinal-type adenocarcinoma, wood dust

## Abstract

**Simple Summary:**

Wood dust exposure is a reported pathogenetic factor for sinonasal intestinal-type adenocarcinoma, particularly in occupational exposure in wood-working industries. This study characterized wood dust exposure and compared the occurrence of sinonasal intestinal-type adenocarcinoma and non-intestinal-type adenocarcinoma between France with predominantly deciduous hardwood forests and Finland with mostly coniferous softwood forests. The findings indicated that sinonasal intestinal-type adenocarcinomas occur much more frequently in France than in Finland and are distinctly more common than non-intestinal adenocarcinomas, while in Finland the reverse is true. This is the first systematic comparison of the occurrence of the two tumor types in countries with distinctly different wood usage and wood dust exposure. It is also the first systematic study on differences in wood dust exposure between sinonasal intestinal-type adenocarcinoma and non-intestinal-type adenocarcinoma. The results provide important epidemiological information on pathogenetic differences between the two tumor types, highlighting the significance of the source of the wood dust.

**Abstract:**

Sinonasal intestinal-type adenocarcinoma is strongly associated with hardwood dust exposure. Non-intestinal-type adenocarcinoma is a rarer and less well-known subtype considered not to be related with wood dust exposure. We determined the relative numbers of these two tumor types in 56 sinonasal adenocarcinoma patients in France and Finland, relating them with carefully assessed wood dust exposure histories. Diagnostic workup including immunohistochemistry for the intestinal markers CDX2 and CK20 indicated that the proportions of the two tumors differed significantly between France and Finland. In Finnish samples non-intestinal adenocarcinomas were more common than intestinal-type adenocarcinomas (12 non-intestinal vs. nine intestinal), while in the French samples the reverse was true (six non-intestinal vs. 29 intestinal). Such remarkably dissimilar occurrence of these tumors in France and Finland presumably reflects different pathogenetic circumstances in the two countries, and perhaps their different patterns of wood dust exposure. In France the main source of wood dust is from hardwoods. In Finland it is derived from softwoods. This is the first systematic comparison of the occurrence of intestinal-type adenocarcinoma and non-intestinal-type adenocarcinoma in two countries with different wood usage. It appears to be the first systematic study on differences in wood dust exposure between intestinal-type adenocarcinoma and non-intestinal-type adenocarcinoma.

## 1. Introduction

The nasal cavity and the sinonasal area function as filters for air entering the lungs. The risk of malignancies in this area is influenced by chemical agents and particles in the air flowing through it. The most important factors affecting the incidence of sinonasal carcinomas (SNC) are various occupational exposures and smoking [1,2,3]. A number of different morphological types of SNC exist, and appear to relate to different exposures to some extent [1].

Sinonasal non-salivary gland-type adenocarcinomas (SNACs) include intestinal-type adenocarcinoma (ITAC) and non-intestinal-type adenocarcinoma (non-ITAC) [4,5]. Histologically ITACs can be subdivided in five morphologic patterns: colonic, papillary, solid, mucinous and mixed [6,7]. ITACs are characterized by histological features typical of intestinal epithelia. ITACs also express intestinal immunophenotypic markers such as cytokeratin 20, intestinal transcription factor CDX-2, MUC-2 [5], and a new marker SATB2 [8]. ITACs usually express cytokeratin 7 typical of airway epithelia [9,10], and they often express chromogranin while the presence of CEA is variable [11]. Molecular genetic studies of ITACs have indicated frequent mutations of *K-RAS* [12] and *TP53* [13,14,15,16].

SNACs that do not exhibit features of ITAC or salivary gland-type adenocarcinomas are designated non-ITACs [10,17,18]. The WHO classification of head and neck tumors (2017) [5] subdivides non-ITACs into high and low-grade types. High-grade non-ITACs are high-grade adenocarcinomas with predominantly solid growth patterns, although glandular and papillary patterns may be detected [5]. In this study all tumors referred to as non-ITACs were of the high-grade type. Low-grade non-ITACs are characterized by a single layer of uniform bland cells forming papillary and glandular structures [4,19,20,21]. Mitotic figures are rare, and there are no atypical mitoses or necrosis. Non-ITACs do not express the above intestine-specific markers, and they are considered to be heterogeneous in origin [17,22]. Recently, a subset of these carcinomas was suggested to represent true seromucinous carcinomas [23].

In epidemiological studies, occurrence of SNACs is strongly linked with exposure to hardwood dusts (particularly beech and oak) and leather dusts [1,3,24,25,26,27]. In contrast, there is very little epidemiological information about the two subtypes, ITACs and non-ITACs, as epidemiological studies are rarely able to address histology in any detail [1,27,28]. The pathology literature, however, associates ITAC, but not non-ITAC, with wood dust exposure [1,3,5,24,25,26,29].

The incidence of SNAC varies between countries and geographical regions, presumably due to differences in dust exposure. In men, the age standardized incidence rates of SNC in France between 2003 and 2007 were 0.8–1.5 per 100,000, and in Finland 0.5 per 100,000 [30], respectively. However, in France, a country with mostly deciduous forests, 36.8% of all SNCs (ICD10 codes C30.0–C31, in 2000–2004) (FRANCIM network, unpublished data) were SNACs. In Finland, where boreal conifer forests predominate 9.3% of all SNCs (C30.0–C31, in 1998–2014) (the Finnish Cancer Registry) were SNACs [31]. Such distributions indicate major differences between France and Finland with regard to SNAC, and likely reflect different characteristics of wood dust exposure as well [3,32]. Figures for the U.S. are close to those observed in Finland, with the incidence rate of SNCs in men being 0.6/100,000 in 2003–2007 [1], and the proportion of SNACs being 12.5% between 1973 and 2006 [33].

In order to investigate the association of SNAC and its subtypes with dissimilar patterns of wood dust exposure, we studied SNACs of 56 patients from France and Finland. In 42 of these cases the occupational history of wood dust exposure has been assessed carefully in a large collaborative study [16,34]. We used immunohistochemical (IHC) markers for intestinal differentiation to determine the relative numbers of ITACs and non-ITACs in these two countries. The study also provides important information about the usefulness of IHC in the diagnostic work-up of SNACs.

## 2. Materials and Methods

### 2.1. Patients and Samples

Formalin-fixed paraffin-embedded tissue samples of sinonasal non-salivary gland type adenocarcinomas (SNACs) were collected from France and Finland as part of a major project on sinonasal cancer called WOODRISK [16,34]. In France, cases were identified in three cancer registries in the areas (départements) of Isère, Somme and Doubs [16]. These areas represent different environments in France, and the occurrence of SNC and the proportion of SNACs in these areas correspond well to those in France in general. In Finland, all incident cases of cancer of the nose and paranasal sinuses (ICD-9 code 160, except 160.1, corresponding to ICD-10 code C30.0, C31) between 1989 and 2002 were identified in collaboration with the Finnish Cancer Registry. All tumor cases included in the study were from Caucasian patients. For all cases, an informed patient consent was obtained. Archival FFPE tissue samples were collected from respective pathology laboratories.

A panel of three pathologists reviewed the histology of all tumor cases of the WOODRISK study (TS, MD, HW) [16,34]. For the present study, representative material of 56 SNACs (both ITACs and non-ITACs) was obtained. A total of 35 cases were from France and 21 from Finland.

The study was approved by the national supervisory committees for medical studies, and received research permissions from the appropriate authorities.

### 2.2. Pathology and Immunohistochemistry

Tissue sections were cut in the pathology laboratory of the Finnish Institute of Occupational Health and stained for hematoxylin and eosin. Immunohistochemical stainings were carried out at the Department of Pathology of Helsinki University Central Hospital using antibodies for CK20, CDX2 and CEA. The tumors were reclassified and the IHC slides interpreted according to the Barnes classification of ITACs [6] by two experienced head and neck pathologists (IL, HW). The intensity of immunohistochemical staining was graded on a scale from 1 to 5, and the extent of the stained tumor area was expressed as a percentage in increments of 10%. An index for immunohistochemical staining was calculated by multiplying the values for intensity and extent.

### 2.3. Work History and Exposure Assessment

Work histories (employment in industry, occupation, periods) of the patients were obtained by interviewing the patient or the next-of-kin. In Finland, the information obtained through interviews on lifelong employment history was supplemented by data from pension records and other statistics [16].

For each case, exposure to wood dust and other risk factors were assessed for the entire work history by one to three experienced industrial hygienists who were blinded to other information of the study. The evaluation of wood exposure was carefully coordinated between the countries [16]. Wood dust exposure estimates were subdivided by wood species and products used (softwood, hardwood, wooden boards). Frequency of exposure (proportion of annual and daily exposure periods/working times) was also estimated. The proportion of each wood type was estimated by industry, occupation and period [16]. Use of softwood such as pine and spruce was more common in Finland than in France. In Finland, the hardwoods oak and beech were used only in the furniture industry and by construction joiners laying wood floors. Most of the wood dust exposure in both countries included a component of mixed wood species. In all cases, the main type of wood exposure (>50%) was recorded. If not enough information was available to determine whether softwood or hardwood was the most prevalent component in wood dust exposure, it was designated as mixed/unspecified [16].

For each job held, the concentration of exposure to wood dust was assessed by industrial hygienists into six categories as described in [16]: unexposed, very low (<0.3 mg/m^3^), low (0.3–1 mg/m^3^), medium 1 (1–2 mg/m^3^), medium 2 (2–5), or high (≥5 mg/m^3^). Quantitative estimations were based on collected national measurement data in France and Finland [16]. A level of exposure was calculated for each job by multiplying the concentration by frequency. Probability of occupational exposure to wood dust was coded in three categories: possible, probable and definite. In all cases, the exposure information was retrospective and obtained only from patients who had developed SNC. The occupational hygienists assessing the patients’ wood dust exposure were not aware of the histology results in the assessed cases.

In this study, all patients with any occupational exposure to wood dust were combined into one group of patients exposed to wood dust. The comparison was between this group and those not exposed. Patients whose probability of exposure was rated as possible (*n* = 2) were left out of the analysis. Of the 56 tumors included in the histological analysis, the information on exposure history was, however, missing in 12 cases.

### 2.4. Statistical Analysis

Comparisons between groups were performed using Fisher’s exact test and McNemars test for paired proportions. A *p*-value of <0.05 was considered statistically significant. Comparisons between (HE) ITACs and (IHC) ITACs with regard to their association to wood dust exposure were made using logistic regression with generalized estimating equations and taking the interdependence of the observations into account.

## 3. Results

### 3.1. Classification of SNACs into ITACs and Non-ITACs by Routine HE Staining Is Not Always Supported by IHC

A total of 56 SNACs was diagnosed using routine HE staining. They included 51 diagnoses of ITAC and five diagnoses of non-ITAC. We then stained all tumors for CK20 and CDX2. The immunohistochemical staining intensity and the extent of the stained area in tumors that were originally classified as ITACs in HE staining are shown in Table 1. To denote diagnoses based on routine staining, the qualifier (HE) is used in this article. A diagnosis based on IHC findings is indicated with the qualifier (IHC). None of the tumors with original diagnosis of (HE) non-ITAC stained positively for CK20 or CDX-2. More than 60% of the tumors with a diagnosis of (HE) ITAC stained for CK20 and CDX-2 with an intensity of 3–5 (33/51 for CK20 and 34/51 for CDX-2). In 45 % (23/51) of the cases more than 70% of the tumor area was stained for CK20, while in 60% (30/50) of the cases similar staining was seen for CDX-2 (Table 1). Intensity of the IHC staining was multiplied with the percentage of the tumor area stained to form an index. As an immunohistochemical criterion for ITAC, the index for either CK20 or CDX2 was required to exceed 70, while those achieving lower indices were considered to be non-ITACs. In reality, however, the distribution between positively staining and negatively staining tumors was quite dichotomous, as can be seen in Table 1. In total, 74.5% (38/51) of the cases filled our immunohistochemical criteria for ITAC, and 60% (30/50) of the cases had both CK20 and CDX-2 indices more than 70.

**Figure 1:**
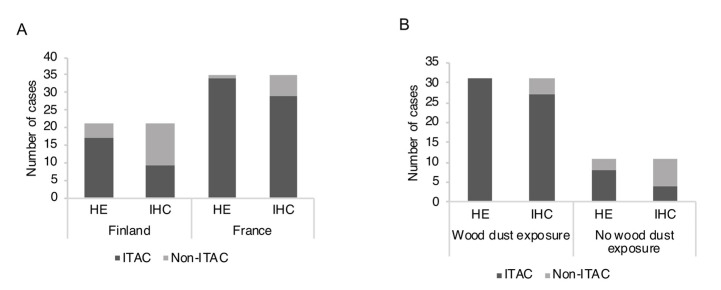
Distribution of ITAC and non-ITAC diagnoses using HE-morphology (HE) and immunohistochemistry (IHC) for CK20 and CDX-2. (**A**) Classification stratified by country of the patients. (**B**) Classification stratified by exposure history. ITAC, intestinal-type adenocarcinoma.

**Figure 2:**
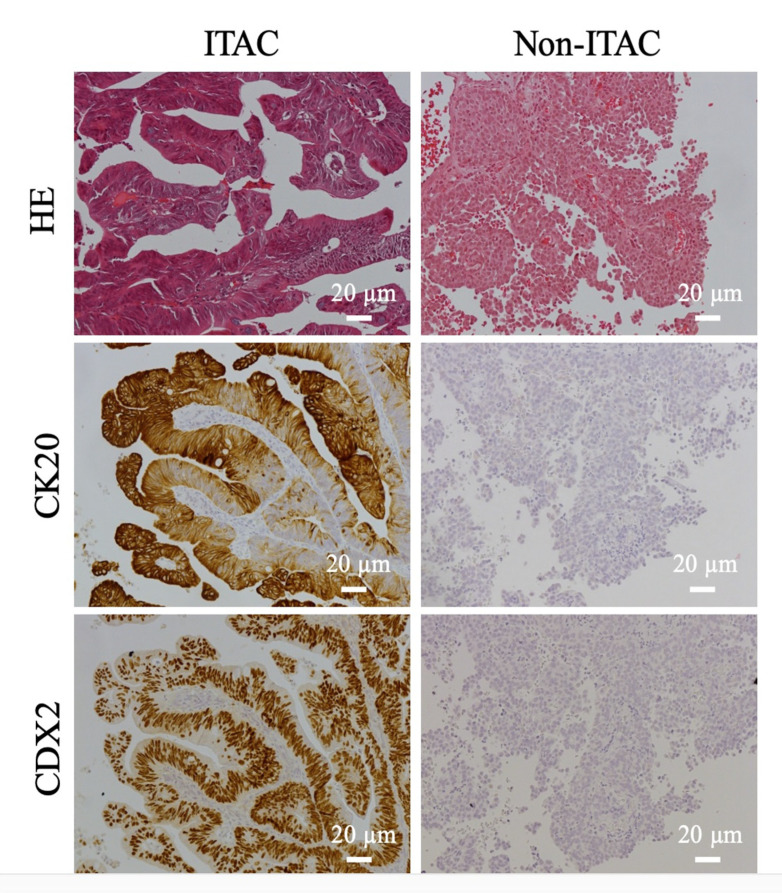
Immunohistochemical stainings of two sinonasal adenocarcinomas with antibodies for the intestinal markers CK20 and CDX2. In the left column, the immunostainings are compatible with ITAC, and on the right with non-ITAC. Scale bars 20 µm. ITAC, intestinal-type adenocarcinoma.

Using these criteria 33/51 ITACs stained for CK20 and 36/51 for CDX-2 (Table 2, Figure 1 and Figure 2). After the IHC evaluation, 13 (HE) ITACs did not meet the criteria for (IHC) ITACs and were thus classified as (IHC) high grade non-ITACs. The difference in the distribution of ITACs and non-ITACs between HE diagnoses and IHC diagnoses was significant (*p* < 0.001). Interestingly, the 13 (IHC) non-ITACs originally classified as (HE) ITACs, displayed many of the histomorphological patterns described by Barnes for ITAC subtypes [6] (Table S1). However, there was a significant difference in the occurrence of the Barnes subtypes in (IHC) ITACs and (IHC) non-ITACs (*p*-value = 0.002). Solid patterns were exclusively seen in non-ITACs, while colonic and mucinous patterns were overwhelmingly seen in ITACs.

### 3.2. Relative Numbers of ITAC and Non-ITAC Differ between France and Finland

Of the 51 ITACs diagnosed with HE staining, 34 were from France and 17 from Finland. After reassessment of ITACs using IHC, 14.7% (5/34) of the French cases were reclassified as (IHC) non-ITACs, while 47.1% (8/17) of the Finnish cases were reclassified as (IHC) non-ITACs (Table 2). The difference between France and Finland in the proportion of revised ITAC diagnoses achieved significance (*p*-value = 0.019). Using IHC as a criterion, the French sinonasal adenocarcinomas were almost entirely ITACs, while the majority of the Finnish sinonasal adenocarcinomas were non-ITACs (Figure 1A). This difference between France and Finland in the distribution of (IHC) ITACs and (IHC) non-ITACs achieved a significant *p*-value of 0.003, while without IHC the corresponding *p*-value was 0.060.

### 3.3. ITAC and Occupational Exposure

The exposure history was known for 42 patients with (IHC) ITAC or (IHC) non-ITAC. A total of 31 patients had been exposed to wood dust at work while 11 had not. Overall, a large majority of the patients who had been exposed to wood dust had (IHC) ITAC (27/31 cases; 87.1%), whereas only a minor proportion of the nonexposed patients had (IHC) ITAC (4/11 cases; 36.3%). The *p*-value for significance of the association of (IHC) ITACs to wood dust exposure was 0.003 (Table 3, Figure 1B). However, if the diagnosis was based on HE staining only, a somewhat smaller proportion of (HE) ITACs, as compared to (IHC) ITACs, was associated with wood dust exposure (31/39 cases; 79.5%). In the nonexposed group, a majority of the tumors were (HE) ITACs, (8/11 cases: 72.7%). The *p*-value for significance of the association of (HE) ITACs to wood dust exposure was 0.0144. Thus, the use of diagnoses based on IHC lowered the amount of ITACs with no occupational exposure history. Comparing (HE) ITACs to (IHC) ITACs with respect to the number of ITACs without wood dust exposure, the odds ratio (OR) was 1.74 (95% confidence interval is 0.86–3.53), *p* = 0.1234, not achieving significance.

Almost all patients with wood dust exposure had been exposed to a minor degree to wood dust from mixed species (93.5%, 29/31). Somewhat surprisingly the main type of wood dust exposure (defined as the exposure >50%) did not have an effect on the proportions of ITAC and non-ITAC cases (*p*-value = 1.0) (Table 3). In France 14/19 (73.7%) of the wood dust exposed (IHC) ITAC patients were mainly exposed to hardwood dust, while the remaining 5/19 (26.3%) French cases were mainly exposed to mixed/unspecified wood dust. None of the French (IHC) ITAC patients were exposed to mainly softwood dust. In Finland 5/8 (62.5%) of the (IHC) ITAC patients were exposed mainly to softwood dust, while 2/8 (25%) were exposed mainly to hardwood dust, and 1/8 (12.5%) mainly to mixed/unspecified wood dust.

The association of (IHC) ITACs to wood dust exposure in general was quite similar in France (86.4% of (IHC) ITACs) and in Finland (88.9%) (Table S2), suggesting that the significance of wood dust exposure to ITAC is rather similar in the two countries.

There was no difference in the relative prevalence of (IHC) ITAC and (IHC) non-ITAC between smokers and nonsmokers (*p*-value = 1.00). In smokers, 70.8% (17/24 cases) of SNACs were ITACs and in nonsmokers the figure was 71.4% (10/14 cases) (Figure S1).

### 3.4. Ki-67 Index and CEA

The Ki-67 labeling index was high (>50%) in 42.1% (16/38 cases) of (IHC) ITACs, while intermediate values (15–50%) were seen in 47.4% (18/38 cases), and the remainder (4/38 cases; 10.5%) had low indices. In non-ITACs a low (<15%) Ki-67 index was more common (6/18 cases; 33.3%) than in (IHC) ITACs (*p*-value = 0.060) (Table S3).

Immunohistochemical staining for CEA was seen in 88.7% (47/53) of the cases. CEA staining was both membranous and cytoplasmic in 64.2% (34/53) and only membranous in 24.5% (13/53) of the cases. There was no statistically significant difference between ITACs and non-ITACs (*p*-value = 0.540) (Table S4).

## 4. Discussion

To investigate the association of SNAC and its subtypes to wood dust exposure patterns we studied 56 SNAC patients from France and Finland. These cases were collected in the context of a large collaborative study on sinonasal cancer and exposure to wood dust [16]. Occupational histories of a large proportion of the patients were carefully assessed by industrial hygienists [16]. We used (IHC) markers of intestinal differentiation (CK20 and CDX-2) in a series of histological SNAC samples from France and Finland to determine as accurately as possible the relative numbers of ITACs and non-ITACs in the two countries, which have very different wood dust exposure patterns. However, it appeared that each marker was also separately able to provide similar results.

Our study revealed that if IHC for intestinal markers was included in the diagnostic work-up, the relative amounts of ITAC and non-ITAC differed significantly between France and Finland. Remarkably, in Finland, non-ITACs were more common than ITACs, while in France non-ITACs were a small minority.

As the histomorphologic criteria of high-grade non-ITAC are rather nonspecific and exclusionary, it is not surprising that the use of markers for intestinal differentiation provided significant information. It is noteworthy that most of the tumors classified as (IHC) non-ITAC also matched one of the histological subtypes of ITAC as illustrated in the Barnes HE-based classification [6]. In addition to being a marker of intestinal differentiation, CDX2 expression is an indicator of the activation of the CDX2 homeobox gene driving intestinal differentiation, not just in ITACs but also in normal tissues. It has been reported by Tilson et al. [35] that some non-intestinal sinonasal malignancies (undifferentiated carcinomas, squamous cell carcinomas, salivary type carcinomas and small cell carcinomas) may express CDX2 to variable extent. In our study, none of the CDX2 positive tumors exhibited features compatible with the CDX2 positive non-intestinal tumors in the study of Tilson et al. [35].

In France traditional hardwoods, such as the oak and the beech, are widely used in woodworking industries, while in Finland conifer softwoods such as the pine and the spruce, and the broadleaf birch are used much more commonly, reflecting the predominant types of trees growing in the respective countries. In Finland, oak and beech are used in very limited quantities, mostly in specific applications such as wood floorings and furniture [3,32]. SNACs are clearly more prevalent in France (about 37% of all SNCs and 46% of all cancers located in the sinuses [FRANCIM network, unpublished data]) compared to Finland (about 9% of SNCs) [31]. This is consistent with a higher degree of occupational exposure to hardwood dust in French industries. As noted earlier, the wood exposure of almost all patients, both in France and in Finland, was to a mixture of hardwoods and softwoods. However, in France the main type of wood in the mixture (defined as >50% of wood) came from hardwoods, while in Finland it came from softwoods.

The differences in wood exposure patterns seen between France and Finland are probably common on the global scale. Interestingly, epidemiologic studies conducted in the U.S. and Canada provide lower risk estimates for SNC in wood working populations as compared to the higher risk levels especially of SNAC observed in numerous studies in Europe [3,36,37]. The percentage of SNACs of all SNC in the U.S. (12.5%) is only slightly higher than that in Finland (9%). Only few U.S. studies have reported on the histological subtypes of SNC, including one that investigated adenocarcinoma [38], while two others focused on squamous cell carcinoma [39,40]. Brinton and coworkers observed a fivefold elevated risk of SNAC associated with employment in the furniture industry in the U.S. [36,38], and reported that 10 of 13 patients with adenocarcinoma were classified as exposed to wood dust [36,38]. Overall, a pooled analysis of 12 case-controlled studies indicated significant positive associations for adenocarcinoma in men, similar to results in Europe, with a dose-response pattern and a 45-fold increased risk estimate for adenocarcinoma among those with the highest wood dust exposures [37,41]. Obviously, more accurate histological information, in particular about the subtypes of adenocarcinoma, would be needed in the U.S. to assess the association between sinonasal adenocarcinoma and occupational wood dust exposure.

In our study, HE and IHC-stained ITACs compared to non-ITACs were seen more often in workers exposed to wood dust. Interestingly, using IHC as a diagnostic criterion, the proportion of non-ITAC cases exposed to wood dust was about 36% (4/11). For comparison, a previous study that included SNCs of all types, reported that 19.8% (33/167) of the cases with squamous cell carcinoma had a history of occupational wood dust exposure [16]. Smoking is a well-known risk factor for squamous cell carcinoma, while ITAC and non-ITAC appeared to be equally common among smokers and nonsmokers, indicating that their relative occurrence is not affected by smoking. Altogether, these data suggests that non-ITAC might have a somewhat stronger association with wood dust exposure than squamous cell carcinoma.

We found some association of non-ITACs with wood dust exposure, but the association was much stronger in ITACs. The respective association of ITAC and non-ITAC with wood dust exposure was remarkably similar in France and Finland when IHC was used in a diagnostic work-up. Interestingly, while the association of ITAC with wood dust exposure was similar in both countries, the incidence of ITAC in France was much higher. This is generally presumed to be due to a higher level of hardwood dust exposure in that country. In France the majority of ITAC patients were exposed to “mainly hardwood” dust, while in Finland ITAC patients usually had a history of “mainly softwood” dust exposure (indicating that >50% of the used wood was softwood). It is noteworthy that five of all 27 (18.5%) our (IHC) ITAC cases with wood dust exposure had been exposed to “mainly softwood” (Table 3). It is, however, not possible to resolve whether ITACs in patients exposed to “mainly softwood” were caused by softwood exposure per se or a minor component of hardwood exposure in their work history.

## 5. Conclusions

Taken together, our results show that the relative frequencies of ITAC and non-ITAC are very different in two countries where the main types of wood used are distinctly different. In France, where hardwoods dominate, ITACs are the overwhelmingly most common type of SNAC. In Finland, where softwoods dominate, non-ITACs are relatively much more frequent. While hardwood exposure seems important in the pathogenesis of ITAC, cases will also emerge after exposure to predominantly softwoods.

## Figures and Tables

**Table 1:** Intensity of staining and percentage of tumor area stained for CK20 and CDX-2.

Intensity of Staining	CK20		CDX-2	
	Finland	France	Finland	France
	*n* (%)	*n* (%)	*n* (%)	*n* (%)
0	7 (41.2)	4 (11.8)	8 (47.1)	5 (14.7)
1	1 (5.9)	1 (2.9)	0 (0)	1 (2.9)
2	0 (0)	5 (14.7)	0 (0)	3 (8.8)
3	3 (17.6)	9 (26.5)	1 (5.9)	6 (17.6)
4	4 (23.5)	13 (38.2)	6 (35.3)	17 (50.0)
5	2 (11.8)	2 (5.9)	2 (11.8)	2 (5.9)
**Tumor Area Stained**				
<10%	7 (41.2)	7 (20.6)	8 (50.0)	5 (14.7)
10–30%	2 (11.8)	3 (8.8)	0 (0.0)	1 (2.9)
40–60%	2 (11.8)	7 (20.6)	0 (0.0)	6 (17.6)
70–100%	6 (35.3)	17 (50.0)	8 (50.0)	22 (64.7)

**Table 2:** SNACs classified with routine staining (HE) or with immunohistochemistry (IHC) for CK20 and CDX-2 in cases from Finland and France.

SNAC	Classification Based on HE	CK20 Positive	CDX-2 Positive	Classification Based on IHC
	*n*	*n* (% ^a^)	*n* (% ^a^)	*n* (% ^a^)
Finland				
*ITAC*	17	8 (47.1)	9 (52.9)	9 (52.9)
*Non-ITAC*	4	0 (0.0)	0 (0.0)	12 (300.0)
France				
*ITAC*	34	25 (73.5)	27 (79.4)	29 (85.3)
*Non-ITAC*	1	0 (0.0)	0 (0.0)	6 (600.0)
Total				
*ITAC*	51	33 (64.7)	36 (70.6)	38 (74.5)
*Non-ITAC*	5	0 (0.0)	0 (0.0)	18 (360.0)

^a^ % indicates the percentage of the HE diagnosis. SNACs, sinonasal non-salivary gland-type adenocarcinomas. ITAC, intestinal-type adenocarcinoma.

**Table 3:** Distribution of the tumors classified with routine stain (HE) or immunohistochemistry (IHC) as ITAC or non-ITAC across different wood dust exposure groups. The percentages refer to the distribution between ITACs and non-ITACs within a group with similar wood exposure and the same method of evaluation.

Wood Dust Exposure	(HE) ITAC	(HE) Non-ITAC	(IHC) ITAC	(IHC) Non-ITAC
	*n* (%)	*n* (%)	*n* (%)	*n* (%)
Yes	31 (100.0)	0 (0.0)	27 (87.1)	4 (12.9)
No	8 (72.7)	3 (27.3)	4 (36.4)	7 (63.6)
**Type of Exposure**				
Mainly Hardwood	18 (100.0)	0 (0.0)	16 (88.9)	2 (11.1)
Mainly Softwood	6 (100.0)	0 (0.0)	5 (83.3)	1 (16.7)
Mixed/unspecified	7 (100.0)	0 (0.0)	6 (85.7)	1 (14.3)

ITAC, intestinal-type adenocarcinoma.

## Data Availability

Data supporting reported results can be obtained from the authors upon request.

## Supplementary Materials

The following are available online at https://www.mdpi.com/article/10.3390/cancers13205245/s1, Figure S1: Distribution of tumors classified as (IHC) ITAC or (IHC) non-ITAC among smokers and non-smokers, Table S1: Distribution of morphologic types (Barnes classification) in tumors classified in HE staining as ITACs, and reclassified based on IHC as ITACs and non-ITACs, Table S2: Association between wood dust exposure and ITACs and non-ITACs in Finland and France classified with HE or IHC, Table S3: Ki-67 labeling index in tumors classified as (IHC) ITAC or (IHC) non-ITAC, Table S4: CEA staining in tumors classified as (IHC) ITAC or (IHC) non-ITAC.
