# Supplementary material for: Occurrence of Sinonasal Intestinal-Type Adenocarcinoma and Non-Intestinal-Type Adenocarcinoma in Two Countries with Different Patterns of Wood Dust Exposure
Source: Cancers (Basel). 2021 Oct 19;13(20):5245. doi: 10.3390/cancers13205245 (PMC8533857; doi:10.3390/cancers13205245)
Supplement: Supplementary file 1 [file cancers-13-05245-s001.zip › cancers-1390045-supplementary.pdf]

Supplementary Materials

Occurrence of Sinonasal Intestinal-Type Adenocarcinoma and Non-Intestinal-Type Adenocarcinoma in Two Countries with Different Patterns of Wood Dust Exposure

Ilmo Leivo, Reetta Holmila, Daniele Luce, Torben Steiniche, Michael Dictor, Pirjo Heikkilä, Kirsti Husgafvel-Pursiainen and Henrik Wolff

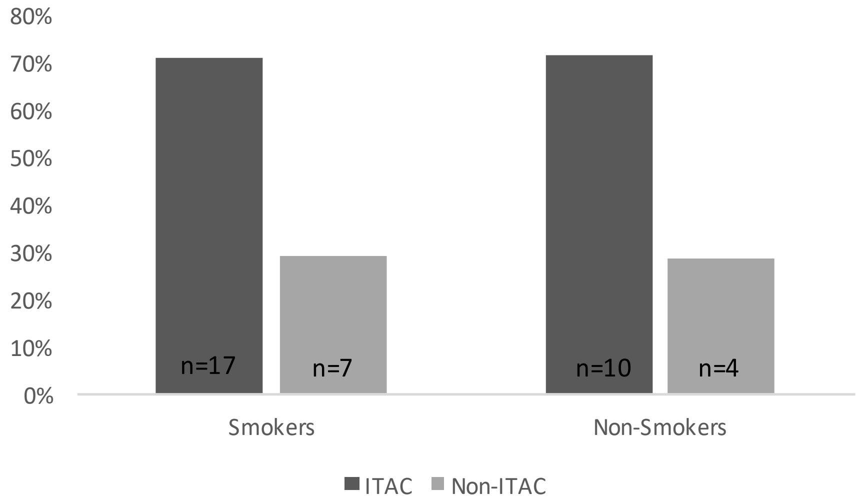

Figure S1. Distribution of tumors classified as (IHC) ITAC or (IHC) non-ITAC among smokers and non-smokers. ITAC, intestinal-type adenocarcinoma; IHC, immunohistochemistry.

Table S1. Distribution of morphologic types (Barnes classification) in tumors classified in HE staining as (ITACs), and reclassified based on (IHC) as ITACs and non-ITACs.

Morphologic Type	(IHC) ITAC *n* (%)	(IHC) non-ITAC *n* (%)
Colonic	15 (39.5)	2 (15.4)
Papillary	8 (21.1)	3 (23.1)
Mucinous	12 (31.6)	1 (7.7)
Mixed	3 (7.9)	2 (15.4)
Solid	0 (0)	3 (23.1)
Other	0 (0)	2 (15.4)
Total	38 (100)	13 (100)

IHC, immunohistochemistry; ITAC, intestinal-type adenocarcinoma.

Table S2. Association between wood dust exposure and ITACs and non-ITACs in Finland and France classified with HE or IHC.

Variable	Finland *n* (%)	France *n* (%)	All *n* (%)
(HE) ITAC			
Wood dust exposure	11 (68.8)	20 (83.3)	31 (79.5)
No exposure	4 (26.7)	4 (16.7)	8 (20.5)
(IHC) ITAC			
Wood dust exposure	8 (88.9)	19 (86.4)	27 (87.1)
No exposure	1 (11.1)	3 (13.6)	4 (12.9)
(HE) non-ITAC			
Wood dust exposure	0 (0)	-	0 (0)
No exposure	3 (100.0)	-	3 (100.0)
(IHC) non-ITAC			
Wood dust exposure	3 (33.3)	1 (50.0)	4 (36.4)
No exposure	6 (66.7)	1 (50.0)	7 (63.6)

ITAC, intestinal-type adenocarcinoma; HE, Hematoxylin and Eosin; IHC, immunohistochemistry.

Table S3. Ki-67 labeling index in tumors classified as (IHC) ITAC or (IHC) non-ITAC.

Ki-67	(IHC) ITAC *n* (%)	(IHC) Non-ITAC *n* (%)
<15%	4 (10.5)	6 (33.3)
15-50%	18 (47.4)	7 (38.9)
>50%	16 (42.1)	5 (27.8)
Total	38 (100)	18 (100)

ITAC, intestinal-type adenocarcinoma, IHC, immunohistochemistry

Table S4. CEA staining in tumors classified as (IHC) ITAC or (IHC) non-ITAC. CEA staining was not available in all tumors.

Variable	(IHC) ITAC *n* (%)	(IHC) Non-ITAC *n* (%)
No CEA	3 (8.1)	3 (18.8)
CEAm and CEAc	25 (67.6)	9 (56.3)
Only CEAm or CEAc	9 (24.3)	4 (25.0)
Total	37 (100)	16 (100)

ITAC, intestinal-type adenocarcinoma; IHC, immunohistochemistry; CEA, carcino-embryonic antigen. CEAm: membranous staining, CEAc: cytoplasmic staining.
